# Antidiarrheal Effect of *Zornia brasiliensis* Vogel (Leguminosae) on Mice Involves Adrenergic Pathway Activation

**DOI:** 10.1155/2021/1385606

**Published:** 2021-02-28

**Authors:** Filipe R. M. B. Oliveira, Sarah R. D. Ferreira, Giulyane T. Aires-Moreno, Anne D. S. Silva, Yuri M. Nascimento, Vicente C. O. Costa, Josean F. Tavares, Marcelo S. Silva, Fabiana A. Cavalcante

**Affiliations:** ^1^Centro de Ciências da Saúde/Universidade Federal da Paraíba, João Pessoa, Paraíba, Brazil; ^2^Programa de Pós-graduação Em Produtos Naturais e Sintéticos Bioativos/Universidade Federal da Paraíba, João Pessoa, Paraíba, Brazil; ^3^Programa de Pós-graduação Em Química e Biotecnologia /Universidade Federal de Alagoas, Maceió, Alagoas, Brazil; ^4^Departamento de Ciências Farmacêuticas/Universidade Federal da Paraíba, João Pessoa, Paraíba, Brazil; ^5^Departamento de Fisiologia e Patologia/Universidade Federal da Paraíba, João Pessoa, Paraíba, Brazil

## Abstract

Several secondary metabolites have been isolated from *Zornia brasiliensis* (Leguminosae), mainly flavonoids. These compounds are known for many pharmacological actions, such as antispasmodic and antidiarrheal. Therefore, we evaluated the antidiarrheal effect of the ethanolic extract obtained from *Zornia brasiliensis* aerial parts (ZB-EtOH_AP_), as well as its underlying mechanisms. Castor-oil-induced diarrhea, fluid accumulation, and intestinal transit (normal and castor oil induced) were performed to assess the antidiarrheal, antisecretory, and antipropulsive activities of the extract. The involvement of opioid and adrenergic pathways was also investigated. ZB-EtOH_AP_ inhibited, in a dose-dependent manner, both total defecation frequency and the number of watery stools. The extract showed no effect on fluid accumulation or normal intestinal transit. On the other hand, when the animals were pretreated with castor oil, the extract decreased the distance traveled by the marker in the small intestine. Investigation of the involvement of opioid and adrenergic systems showed that the pharmacological potency of the extract did not change in the presence of naloxone, but it was reduced in the presence of yohimbine. The data indicate that *Zornia brasiliensis* has an antidiarrheal effect due to inhibition of the intestinal motility through adrenergic pathway activation.

## 1. Introduction

Diarrhea is an issue not only in the developing world but also in the western world. Diarrhea is considered the most common worldwide cause of death of children under 5 years of age [1]. In this context, the World Health Organization (WHO) has encouraged studies for the treatment and prevention of diarrheal diseases based on traditional medicinal practices and use of natural resources [2].

*Zornia brasiliensis* Vogel is a herbaceous plant popularly known as *urinana*, *urinária*, or *carrapicho*. It has been used in folk medicine as a diuretic and for venereal disease treatment [3]. According to phytochemical analysis of the ethanolic extract obtained from the aerial parts of *Z. brasiliensis* (henceforth ZB-EtOH_AP_), several secondary metabolites have been isolated from this species, including saponins (soyasaponin IV and *A*_3_) and terpenes (dihydromelilotosideo and roseosideo), among others. However, extracts obtained from *Z. brasiliensis* are mainly known for containing considerable amounts of flavonoids, such as 5-hydroxy-7-methoxyflavone, 7,4-dimethoxy-isoflavone, 7-methoxyflavone, and 5,7-dimethoxyflavone [4–6].

Plants with high amounts of flavonoids have been used to treat a wide variety of diseases, including diarrhea [7]. For instance, a mixture of flavonoids obtained from *Malus pumila* leaves exhibited an antidiarrheal effect [8]. Similarly, isolated flavonoids, such as quercetin and rutin, have been shown to attenuate diarrhea symptoms by inhibiting intestinal muscle contractility, enhancing intestinal motility, and reducing fluid intraluminal accumulation in the gut lumen, as evidenced in different experimental studies [9].

Therefore, considering that ZB-EtOH_AP_ presents a phytochemical characterization that shows its high concentration of flavonoids and that these compounds may have antidiarrheal activity, ZB-EtOH_AP_ was chosen for quantification of its main components, evaluation of antidiarrheal effect, and its underlying mechanisms. As the main hypothesis of the work, we have that the extract will present an antidiarrheal effect by inhibiting the production of intestinal fluids and decreasing peristalsis.

## 2. Materials and Methods

### 2.1. Plant Material and Crude Extract Preparation

Aerial parts of *Zornia brasiliensis* Vogel (Leguminosae) were collected in the municipality of Serra Branca (07°29′46 ″S and 36°44′36″ W, 712 m altitude), state of Paraíba (PB), Brazil, in March 2016. It was identified by botanist Dr. José Iranildo Miranda de Melo, Universidade Estadual da Paraíba. Voucher specimens were deposited in Herbarium Arruda Câmara (HACAM), Campus I of Universidade Estadual da Paraíba, verification code 1862. Access registration in the National Management System of Genetic Patrimony and Associated Traditional Knowledge (SISGEN, Curitiba, Brazil) was obtained under code ADD107E.

Aerial parts of *Z. brasiliensis* were dried and crushed in a knife mill. The powder (5 kg) was subjected to extraction with ethanol (EtOH) 95%, by maceration, in proportion of 1 : 3 [w (kg)/v (L)] at room temperature. The extractive solution was collected every 72 hours, this collection, and the replacement of the solvent were repeated four times under the same powder. Then, the extractive solution was concentrated under reduced pressure in a rotary evaporator [5]. Flavonoids 7-methoxyflavone and 5,7-dimethoxyflavone were used as chemical markers for analysis and quantification, once they were isolated from ZB-EtOH_AP_ [5, 6].

### 2.2. Analysis by High-Performance Liquid Chromatography

A Shimadzu Prominence chromatograph equipped with an LC-20AT solvent pump, a SIL-20A self-injector, a DGU-20A degassing system, an SPD-M20A diode arrangement detector, a CTO-20A furnace, and a CBM-20A controller system was used for the chemical analysis. A Kromasil^®^ C4 column (250 mm × 4.6 mm ID, 3.5 *μ*m) and a Kromasil^®^ C4 precolumn (4.6 mm ID *x* 3.0 mm, 3.5 *μ*m) were used. Analyses of the data obtained by HPLC-DAD were performed using the software Lab Solutions^®^ (Shimadzu). The mobile phase was composed of water (0.1% formic acid) and acetonitrile (1 : 1, v/v) in an isocratic mode for 23 min at a flow rate of 0.45 mL/min, temperature of 40°C. The injection volume was 10 *μ*L, and the detection was performed at 254 nm. Samples were filtered on 0.45 *μ*m nylon membranes (Tedia). Five solutions with different masses were prepared with 7-methoxyflavone (2.7–3.9 *μ*g) and 5,7-dimethoxyflavone (1.2–2.0 *μ*g) and injected in triplicate to obtain the respective linear regression equations, as well as the determination coefficients. Both markers were quantified in 20.0 *μ*g of the crude extract. The limits of detection (LOD) and quantification (LOQ) were determined based on what is recommended by the Resolução da Diretoria Colegiada (RDC) nº 166, June 24, 2017 [10].

### 2.3. Animals

A total of 264 two-month-old male and female Swiss mice (*Mus muscullus*) weighing 25–35 g, obtained from the bioterium Professor Thomas George of Instituto de Pesquisa em Fármacos *e* Medicamentos (IPeFarM)/UFPB, were used. Prior to the experimental protocols, the animals were kept under a balanced dietary control (Labina^®^), with free access to water, in a temperature-controlled room (21 ± 1°C), and were daily exposed to a 12-hour light/dark cycle (light period from 6 am. to 6 pm.). On the day of the experiments, the animals were transferred to the experimental facility and acclimated for 30 min until the experimental protocols were initiated. After acclimatization, the animals were randomly assigned to the different groups. All experimental procedures were conducted following the principles of animal care Guidelines for the ethical use of animals in applied etiology studies [11] and approved by the Ethics Committee on Animal Use/CEUA (Certificate Nos. 0605/12 and 3206/13) of Universidade Federal da Paraíba.

### 2.4. Drugs

Unless otherwise stated, all compounds were from Sigma-Aldrich Company Ltd. (Brazil). Sodium chloride (NaCl) was purchased from Química Moderna (Brazil). Loperamide was from Janssen-Cilag^®^ (Brazil); castor oil was from Farmax^®^ (Brazil), and naloxone chloride was purchased from Cristália^®^ (Brazil).

ZB-EtOH_AP_ was solubilized in Cremophor EL^®^ (3%), dissolved in distilled water to the concentration of 50 mg/mL, and rediluted in distilled water as required for each experimental protocol. 7-Methoxyflavone (94%) and 5,7-dimethoxyflavone (94%) were isolated from aerial parts of *Z. brasiliensis*. The purities of these substances were obtained by mass spectrometry.

### 2.5. ZB-EtOH_AP_ Dose Scheme

The stock solution (50 mg/mL) was diluted in distilled water to lower concentrations as needed. As an initial screening for the extract effect, the mice were treated with doses of 31.2, 62.5, and 125 mg/kg, with the exception of the normal intestinal transit protocol, in which the animals did not receive the diarrhea-inducing agent (see below). Depending on the observed effect in each experimental protocol, the doses could be increased by multiples of two, up to 500 mg/kg, or decreased by half until no more effects of the extract were observed.

### 2.6. Effect of ZB-EtOH_AP_ on Castor-Oil-Induced Diarrhea

Male and female mice were divided into three groups (*n* = 6) that received saline 0.9% plus Cremophor^®^ (10 mL/kg, *p.o*., negative control), loperamide (10 mg/kg, *p.o*., positive control), or ZB-EtOH_AP_ (different doses, *p.o*.). After 30 min of treatment, castor oil was administrated (10 mL/kg, *p.o*.). The animals were separated and placed into individual cages lined with white paper. Then, the animals were inspected for the number of stools and its consistency for 4 h, which were classified into solid or liquid. The total number of stools and the number of liquid episodes were determined. [12].

### 2.7. Effect of ZB-EtOH_AP_ on Castor-Oil-Induced Intestinal Fluid Accumulation

After a 24-hour fasting period, the male and female mice (*n* = 6) were treated with saline 0.9% plus Cremophor^®^ (10 mL/kg, *p.o*., negative control group), loperamide (10 mg/kg, *p.o*., positive control group) or ZB-EtOH_AP_ (different doses, *p.o*.). Thirty min later, castor oil (2 mL/animal, *p.o*.) was administrated and, after 30 min of the administration, the mice were euthanized by cervical dislocation. Their small intestine was carefully removed, preventing any content leakage, and immediately weighed [13]. The results corresponding to the measurement of intestinal fluid accumulation were expressed as (Pi/Pm) × 1000, where Pi is the weight of the intestine and Pm is the weight of the animal in grams [13].

### 2.8. Effect of ZB-EtOH_AP_ on Normal Intestinal Transit

The male and female mice were divided into three groups (*n* = 6) and, after a 12-hour fasting period, were treated with saline 0.9% plus Cremophor^®^ (10 mL/kg, *p.o*., negative control group), atropine (2 mg/kg, *p.o*., positive control group), or ZB-EtOH_AP_ (different doses, *p.o*.). Thirty min later, activated charcoal 5% (10 mL/kg) solubilized in carboxymethylcellulose (0.5%) was administered, and after 30 min of the administration, the animals were euthanized by cervical dislocation. The abdominal cavity was opened, and the small intestine was removed. In order to measure the intestinal transit, the total length of the small intestine (distance from the pylorus to ileocecal valve) and the distance traveled by the marker were measured and expressed in percentage [14] as follows: IT = DT/TL *x* 100, where IT is the intestinal transit, DT corresponds to the distance traveled by the charcoal, and TL stands for the total length [15].

### 2.9. Effect of ZB-EtOH_AP_ on Castor-Oil-Induced Intestinal Transit

The same procedures described in “*Effect of ZB-EtOH*_*AP*_*on normal intestinal transit”* were applied in this phase, with the exception that castor oil (10 mL/kg, *p.o*.) was administrated 30 min before the activated charcoal.

### 2.10. Investigation of the ZB-EtOH_AP_ Antidiarrheal Action Mechanism

To evaluate opioid system involvement, animals were divided into four groups: one group was treated orally with 0.9% saline solution plus Cremophor^®^ (10 mL/kg, negative control), two other groups were treated subcutaneously (s.c.) with morphine (10 mg/kg, positive control), which is an opioid agonist, and the fourth group received ZB-EtOH_AP_ (different doses, p.o.). One of the groups that received morphine and the group that received ZB-EtOH_AP_ were pretreated with naloxone (2 mg/kg, s.c.), an opioid antagonist, 30 min before the administration of morphine or extract. The same method was used to assess the adrenergic pathway, but animals were treated with *α*_2_-agonist clonidine (0.1 mg/kg, p.o., positive control) instead of morphine and the antagonist yohimbine (1 mg/kg, i.p.) instead of naloxone [16].

After 30 min of the treatments described above, animals were treated with castor oil (10 mL/kg, p.o.). The protocol was followed as mentioned before in “*Effect of ZB-EtOH*_*AP*_*on normal intestinal transit*”.

### 2.11. Statistical Analysis

All results were expressed as the mean ± standard error of the mean (S.E.M.) and were statistically analyzed using Student's t-test or one-way variance analysis (ANOVA) followed by Bonferroni's posttest, as needed. The difference between the means were considered significant when *p* < 0.05. All data were analyzed using the GraphPad Prism^®^ software, version 7 (GraphPad Software Inc., San Diego, CA, USA). The inhibitory effect of each extract dose was calculated as the difference, in percentage of the mean ± S.E.M. values of each group out of the mean ± S.E.M. value of the negative control group. The maximum inhibitory effect of the extract (*E*_max_) was used as an efficacy parameter. The dose of a drug or extract that produces 50% of its own maximal response (ED_50_) was calculated by nonlinear regression analyses and was used as a potency parameter.

## 3. Results

### 3.1. Chemical Analysis

**Figure 1:**
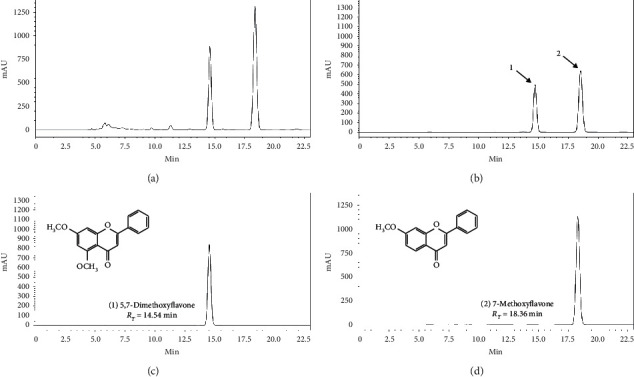
HPLC-DAD analysis of the crude ethanol extract of *Zornia brasiliensis*. (a) Chromatogram of the extract of *Z. brasiliensis*. (b) Chromatogram of the mixture of the chemicals standards 5,7-dimethoxyflavone (1) and 7-methoxyflavone (2). (c) Chromatogram of the chemical standard 5,7-dimethoxyflavone. (d) Chromatogram of the chemical standard 7-methoxyflavone. *R*_*T*_ = retention time.

After the extraction process, the crude ethanolic extract yielded 11% of the weight of the initial plant material. The compounds quantified in ZB-EtOH_AP_ were 7-methoxyflavone (14.65%) and 5,7-dimethoxyflavone (7.44%). Both flavonoids were quantified by high-performance liquid chromatography coupled to a diode array detector (HPLC-DAD). These two phenolic compounds are the most abundant in the crude ethanolic extract, representing 22.09% of all secondary metabolites. Limits of detection (LOD) were 0.19 and 0.33 *μ*g, and limits of quantification (LOQ) were 0.58 and 1.00 *μ*g for 7-methoxyflavone and for 5,7-dimethoxyflavone, respectively (Figure 1).

### 3.2. Effect of ZB-EtOH_AP_ on Castor-Oil-Induced Diarrhea

**Figure 2:**
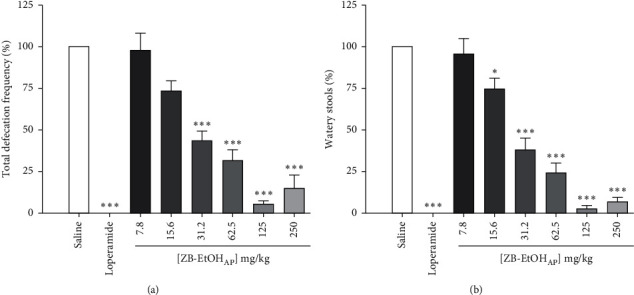
Effect of ZB-EtOH_AP_ on castor-oil-induced diarrhea in mice. (a) Percentage of total defecation frequency and (b) percentage of watery stools. Columns and bars represent the percentage of the mean ± SEM, respectively (*n* = 6). ANOVA “one-way” followed by Bonferroni's posttest: ^*∗*^*p* < 0.05 and ^*∗∗∗*^*p* < 0.001 (saline *vs.* loperamide/ZB-EtOH_AP_).

The extract inhibited, in a dose-dependent manner, both the total defecation frequency (ED_50_ = 31.9 ± 6.5 mg/kg, *R*^2^ = 0.976 ± 0.008, Figure 2(a)) and the number of watery stools (ED_50_ = 28.6 ± 5. mg/kg, *R*^2^ = 0.985 ± 0.006, Figure 2(b)). ZB-EtOH_AP_ maximum effect (*E*_max_) values were obtained with the 125 mg/kg dose for both defecation frequency and watery stools (*E*_max_ = 95.1 ± 2.5 and 97.3 ± 1.7%, respectively) and were similar to loperamide (*E*_max_ = 100%), which produced maximal inhibition of both total defecation and liquid stools.

### 3.3. Effect of ZB-EtOH_AP_ on Castor-Oil-Induced Intestinal Fluid Accumulation

**Figure 3:**
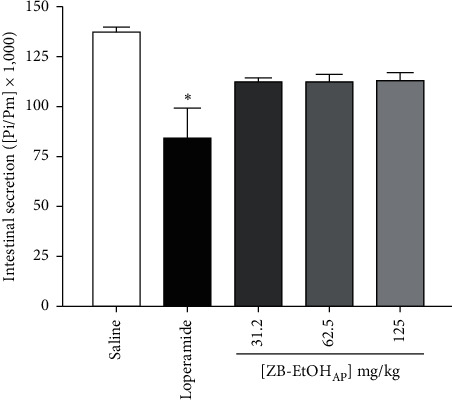
Effect of ZB-EtOH_AP_ on castor-oil-induced intestinal fluid accumulation in mice. Columns and bars represent the mean ± SEM of intestinal fluid secretion, respectively (*n* = 6). ANOVA “one-way” followed by Bonferroni's posttest: ^*∗*^*p* < 0.05 (saline *vs.* loperamide/ZB-EtOH_AP_).

ZB-EtOH_AP_ (31.2, 62.5 and 125 mg/kg) had no significant effect on the intestinal secretion, differently from loperamide, which inhibited fluid secretion (*E*_max_ = 39.0 ± 11.0%) (Figure 3).

### 3.4. Effect of ZB-EtOH_AP_ on Normal Intestinal Transit

**Figure 4:**
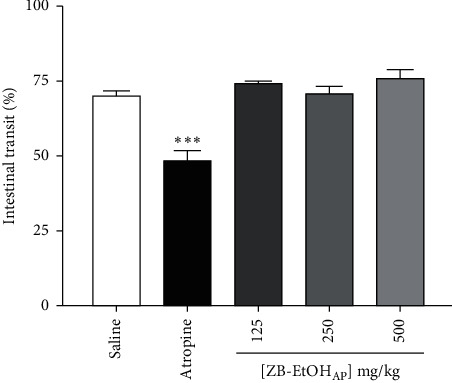
Effect of ZB-EtOH_AP_ on normal intestinal transit in mice. Columns and bars represent the percentage of the mean ± SEM of the distance traveled by the marker, respectively (*n* = 6). ANOVA “one-way” followed by Bonferroni's posttest: ^*∗∗∗*^*p* < 0.001 (saline *vs.* atropine/ZB-EtOH_AP_).

ZB-EtOH_AP_ (125, 250 and 500 mg/kg) did not inhibit the distance traveled by the marker in a context of normal intestinal transit. However, atropine showed an inhibitory effect of 31.1 ± 5.2% on the distance traveled by the activated charcoal in mice intestine when compared with the negative control group (Figure 4).

### 3.5. Effect of ZB-EtOH_AP_ on Castor-Oil-Induced Intestinal Transit

**Figure 5:**
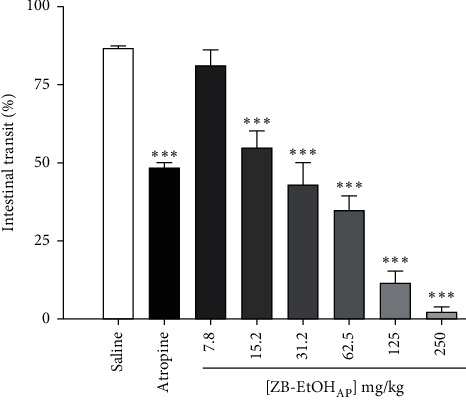
Effect of ZB-EtOH_AP_ on castor-oil-induced intestinal transit in mice. Columns and bars represent the percentage of the mean ± SEM of the distance traveled by the marker, respectively (*n* = 6). ANOVA “one-way” followed by Bonferroni's posttest: ^*∗∗∗*^*p* < 0.001 (saline *vs.* atropine/ZB-EtOH_AP_).

ZB-EtOH_AP_ (7.8–250 mg/kg) inhibited, in a dose-dependent manner (*R*^2^ = 0.891 ± 0.027), castor-oil-induced intestinal transit (ED_50_ = 20.0 ± 4.6 mg/kg). The maximum effect of ZB-EtOH_AP_ was obtained with a dose of 250 mg/kg (*E*_max_ = 97.6 ± 2.0%). In addition, atropine presented an inhibitory effect of 44.0 ± 2.0% on the distance traveled by the marker on castor-oil-induced intestinal transit when compared with the group that received saline (Figure 5).

### 3.6. Evaluation of Opioid System Involvement

**Figure 6:**
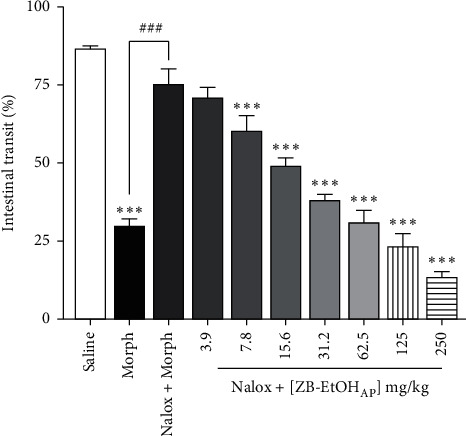
Effect of ZB-EtOH_AP_ on castor-oil-induced intestinal transit in mice in the presence of naloxone. Columns and bars represent the percentage of the mean ± SEM of the distance traveled by the marker, respectively (*n* = 6). ANOVA “one-way” followed by Bonferroni's posttest: ^*∗∗∗*^*p* < 0.001 (saline *vs.* morphine/naloxone + ZB-EtOH_AP_); ^*###*^*p* < 0.001 (morphine *vs.* naloxone + morphine).

An inhibitory effect of 65.9 ± 3.0% on castor-oil-induced transit was observed in the animals treated with morphine. However, when naloxone was administered, this inhibitory effect was thoroughly reversed (Figure 6).

Although the maximum effect obtained with a dose of 250 mg/kg was slightly reduced to 84.5 ± 2.2%, the inhibitory potency of ZB-EtOH_AP_ on castor-oil-induced transit (ED_50_ = 20.0 ± 4.6 mg/kg, Figure 5) did not change in the presence of naloxone (ED_50_ = 10.9 ± 2.6 mg/kg, *R*^2^ = 0.961 ± 0.007, Figure 6).

### 3.7. Evaluation of Adrenergic System Involvement

**Figure 7:**
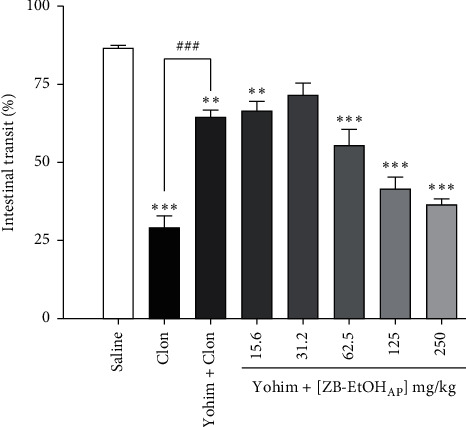
Effect of ZB-EtOH_AP_ on castor-oil-induced intestinal transit in mice in the presence of yohimbine. Columns and bars represent the percentage of the mean ± SEM of the distance traveled by the marker, respectively (*n* = 6). ANOVA “one-way” followed by Bonferroni's posttest ^*∗∗∗*^*p* < 0.001 (saline *vs.* clonidine/yohimbine + clonidine/yohimbine + ZB-EtOH_AP_); ^*###*^*p* < 0.001 (clonidine *vs.* yohimbine + clonidine).

Clonidine reduced the distance traveled by the marker in 66.6 ± 4.6%. As expected, this effect was diminished when the mice were treated with yohimbine (Figure 7). The effect of ZB-EtOH_AP_ was also significantly attenuated in the presence of yohimbine (*E*_max_ = 57.8 ± 2.1%), and its potency was reduced (ED_50_ = 67.9 ± 14.2 mg/kg, *R*^2^ = 0.959 ± 0.018) when compared with the extract potency in the absence of yohimbine (ED_50_ = 20.0 ± 4.6 mg/kg, Figure 5).

## 4. Discussion

Based on the chemotaxonomic criterion, this study aimed at assessing the antidiarrheal effect of *Zornia brasiliensis* on mice and its possible underlying mechanism by evaluating its effect on intestinal secretion and motility. ZB-EtOH_AP_ showed an antidiarrheal effect due to the activation of the adrenergic system, which leads to inhibition of gut motility.

As expected, the quantification of secondary metabolites showed that ZB-EtOH_AP_ is composed mainly of flavonoids, being 7-methoxyflavone and 5,7-dimethoxyflavone present in higher concentrations. These compounds, together, represent more than one-fifth of the metabolites contained in the extract. It has been reported that extracts that have high concentrations of these compounds present vasorelaxant and antispasmodic effects [17]. In addition, natural products known for containing these substances have been used to treat various gastrointestinal disorders [18].

Castor oil is widely used for the screening of drugs with a possible antidiarrheal property [19]. This pharmacological tool causes diarrhea due to the increase of intestinal fluid contents, through ricinoleic acid formation, which affects electrolytes and water transport [20]. Castor oil causes a reduction in intestinal absorption of Na^+^ and K^+^ and decreases Na^+^/K^+^-ATPase activity in the small intestine and colon through its active metabolite, resulting in changes of electrolyte permeability, leading to diarrhea [21, 22]. When tested against castor-oil-induced diarrhea in mice, ZB-EtOH_AP_ produced a remarkable antidiarrheal effect, evidenced by both stool frequency reduction and decrease of the liquid stools number, just like loperamide, one of the standard antidiarrheal agents [23]. In addition, we deem the extract to have a surprisingly interesting antidiarrheal action profile since the extract still presented antidiarrheal effect at doses as low as 31.2 mg/kg and a low ED_50_ value, especially considering that it is a crude extract rather than the isolated substance, which highlights the potential of this species for a possible therapeutic use.

Once the antidiarrheal effect of ZB-EtOH_AP_ was confirmed, it became necessary to evaluate which mechanism was involved in this action, as this effect can occur by reduction of intestinal motility or intestinal secretion inhibition [24]. The intestinal epithelium absorbs and secretes large volumes of fluid. Intestinal fluid secretion involves ionic transport from the blood into the intestinal lumen [25]. For example, Cl^−^ is transported through channels on the enterocyte apical membrane, which include the cAMP-gated channel CFTR (cystic fibrosis transmembrane conductance regulator) [26]. Drugs that are able to inhibit those channels may be effective against secretory diarrheas [27]. Thereunto, the effect of ZB-EtOH_AP_ on castor-oil-induced intestinal fluid accumulation was investigated. However, the extract did not decrease intestinal secretion, partially rejecting our initial hypothesis. On the other hand, the standard drug loperamide significantly decreased the intestinal content, provided that it acts on *μ*-opioid receptors and performs antisecretory actions. Therefore, we conclude that the antidiarrheal activity of ZB-EtOH_AP_ does not depend on intestinal secretion inhibition.

It was still necessary to evaluate the effect of ZB-EtOH_AP_ on the intestinal tone; thus, its effect on intestinal motility was investigated. Activated charcoal (marker) has been used since the 50s as a tool to evaluate laxative effect [28]. This method is an indicator of the maximum distance traveled by the marker and consists of marker administration and assessment of its course in the small intestine over a period of time [16]. As a result, we observed that the extract decreased the distance traveled by the marker in castor oil presence, which was not observed in the evaluation of the normal traffic model. These results suggest that the extract antidiarrheal effect occurs due to the decrease of intestinal motility during diarrhea, confirming our initial hypothesis. However, in a healthy intestine, the extract did not have a constipating effect, even when higher doses of the extract were administered. In a different manner, atropine, the standard drug, inhibited both normal and castor-oil-induced transit. Atropine is a muscarinic antagonist that competes for the same binding site of the acetylcholine neurotransmitter. Its actions include reduction of tone, amplitude, and frequency of intestinal peristaltic contractions [29].

Controlling the contractile activity is complex, with contributions from the intestinal layers themselves, as well as local nerves of the enteric nervous system, the autonomic nervous system, and circulating hormones. Gastrointestinal motor tone is regulated through multiple physiological mediators, such as acetylcholine, histamine, substance P, cholecystokinins, prostaglandins, and 5-hydroxytryptamine [30–33]. The opioid pathway is important in the intestinal activity modulation. There are three different types of opioid receptors: *δ*, *κ*, and *μ*, and all of them belong to the *G*_i/o_ protein coupled receptor family. Activation of these receptors by agonists, such as morphine, leads to the inhibition of adenylyl cyclase, which leads to the reduction of neuronal excitability and release of neurotransmitters, such as acetylcholine, resulting in decrease of intestinal motility [34].

In the investigation of the involvement of the opioid pathway, although the maximum effect of ZB-EtOH_AP_ was minimally reduced, and the inhibitory potency of the extract on castor-oil-induced transit did not change in the presence of naloxone when compared to its effect in absence of naloxone. This means that the pharmacological potency of the extract was not altered in the presence of the opioid antagonist. Therefore, we can conclude that the modulation of opioid receptors is not involved in the action mechanism of ZB-EtOH_AP_, unlike a considerable amount of other drugs that are currently used for the management of diarrhea [35]. This might attribute an interesting characteristic to the extract, considering that its metabolites act in different pathways, possibly being another alternative in the therapeutic arsenal for treatment, for example, of patients who do not respond to loperamide.

Another important pathway in the regulation of intestinal activity is the adrenergic system, which modulates the release of stimulatory neurotransmitters. Alpha-2 (*α*_2_) adrenergic receptors are extensively distributed in the gastrointestinal tract, playing a crucial role in the release of neurotransmitters such as acetylcholine, which regulates the intestinal smooth muscle tone [36, 37]. Clonidine, an *α*_2_-adrenergic agonist, may be useful for the treatment of diarrhea in diabetic patients and in patients with diarrhea due to opioid withdrawal [38]. It stimulates absorption, inhibits secretion, and delays intestinal transit [39, 40]. In the investigation of the adrenergic pathway involvement, we observed that both efficacy and potency of ZB-EtOH_AP_ were reduced in the presence of yohimbine, an adrenergic antagonist. Thus, we concluded that ZB-EtOH_AP_ presents metabolites that positively modulate the adrenergic pathway, resulting in delay of intestinal transit and antidiarrheal effect.

## 5. Conclusions

This study showed that the ethanolic extract of *Zornia brasiliensis* contains high concentrations of flavonoids. In addition, the extract presents an antidiarrheal effect due to the inhibition of intestinal motility, mediated by the activation of the adrenergic pathway. Further studies are necessary to entirely clarify the antidiarrheal mechanisms of the extract, since other pharmacological targets might be involved in the antipropulsive effect of the extract, such as calcium channels.

## Data Availability

All the results used in this work to support the conclusions of this study are included in the article.
